# The impact of AI integration in project preparation in education course on pre-service teachers’ innovativeness, AI anxiety, attitudes, and acceptance

**DOI:** 10.1186/s40359-025-03647-3

**Published:** 2025-11-24

**Authors:** Mustafa Sat

**Affiliations:** Educational Sciences, Faculty of Education, Kırıkkale University, Kırıkkale, Türkiye

**Keywords:** Research literacy, Individual innovativeness, AI anxiety, AI attitudes, AI acceptance, AI tools

## Abstract

The rapid integration of artificial intelligence (AI) in education has transformed teacher education programs. This study examined the effects of incorporating AI tools into a *Project Preparation in Education* course for pre-service teachers, focusing on individual innovativeness, AI anxiety, AI attitudes, and AI acceptance. Using a single-group pre-test and post-test design, validated scales were administered to 34 pre-service teachers before and after the intervention. Paired-samples *t*-tests with Holm–Bonferroni corrections revealed significant improvements in AI attitudes (*g*ₐ_v_ = 0.64) and AI acceptance (*g*ₐ_v_ = 0.70). Individual innovativeness increased slightly, primarily in opinion leadership subdimension (*g*ₐ_v_ = 0.35), whereas AI anxiety showed no significant change (*g*ₐ_v_ ≈ 0.05). These medium-to-large gains. underscore the potential of guided and AI-supported instruction to enhance teachers’ readiness for AI integration across the stages of the academic writing process. However, the absence of a control group limits causal inference, and the results should be interpreted as correlational evidence of change. Overall, the study highlights both the promise and boundaries of short-term AI interventions in teacher education, offering implications for curriculum design and professional development in AI-driven learning environments.

**Supplementary Information:**

The online version contains supplementary material available at 10.1186/s40359-025-03647-3.

## Introduction

The rapid evolution of Artificial Intelligence (AI) technologies has sparked a transformative shift in education, fundamentally reshaping how learning is personalized and how teaching practices are delivered accordingly. AI in education has emerged as a significant field, offering tailored learning experiences and enhancing administrative efficiency [1]. AI applications include adaptive learning systems, intelligent tutoring, assessment tools, and predictive analytics [2, 3]. These technologies improve student engagement, offer 24/7 access to learning, and provide real-time feedback [4]. However, challenges remain, including the need for educator training, curriculum adaptation, and ethical concerns [5].

AI has been widely adopted in modern education, evolving from computer-assisted technologies to advanced systems such as humanoid robots and chatbots. Its adoption has transformed the educational landscape by enhancing administrative functions, teaching activities, and personalized learning experiences [1, 6, 7]. AI is transforming education by enabling more personalized, responsive, and data-driven instruction, moving classrooms beyond a one-size-fits-all model so educators can devote more time to substantive teaching and timely interventions [6–10]. However, AI’s transformative potential heavily relies on educators’ preparedness and enthusiasm—especially among pre-service teachers—whose training and competence shape effective classroom adoption [9, 10]. Their openness to new ideas, adaptability, and eagerness to harness AI’s potential are crucial for integrating these technologies into classrooms [8, 11].

Embracing AI and utilizing its capabilities requires shifting from traditional strategies to learner-centered approaches [8]. The intentions and attitudes of pre-service teachers toward AI influence its potential widespread use in classrooms [7]. Their willingness to change, adaptability, and dedication to using AI for educational enhancement will determine the success of AI adoption in teaching practices [12]. Studies show that students with a stronger understanding of AI tend to hold more positive perceptions of AI and creativity, exhibiting greater acceptance of its integration into classroom settings [13]. Furthermore, student-AI collaboration can significantly affect creativity, expressiveness, and practical application in learning tasks, contingent on students’ attitudes and skill levels [14].

Although AI tools have become a widely utilized technology across various academic and professional domains [15], existing scholarship highlights the effective integration of AI in education depends on students’ ethical awareness and the development of reliable AI detection tools [16]. OpenAI’s ChatGPT is one of the most widely used AI tools, recognized for its versatility in generating text, summarizing information, answering questions, and supporting students in a range of academic and professional tasks [17, 18]. For example, it enhances writing skills by correcting grammatical errors, improving clarity, and ensuring consistency through real-time feedback [19]. AI tools offer significant potential for scientific/academic research and writing. It can assist researchers in tasks such as literature reviews, content summarization, and manuscript editing, potentially enhancing productivity and efficiency [20]. It can also support various aspects of research, from formulating questions to drawing conclusions [21]. However, researchers foreground some concerns about the use of AI technology in education. They highlights some of these concerns as academic integrity, over-reliance on AI, and the need for clear usage guidelines [22]. Data privacy is another important concern, because AI systems often rely on the collection and analysis of extensive sensitive student data [11]. In addition, it is stated that depending on AI for personalized learning could reduce the human connection and emotional insight that define the teacher-student relationship [23]. The unequal distribution of digital resources across different regions and socioeconomic groups limits access to AI-enhanced education, thereby reinforcing existing inequalities [24].

Furthermore, biased AI in education leads to unequal outcomes, raising ethical concerns about its transparency and accountability [25]. Finally, the swift pace of technological innovation often outstrips the professional development infrastructure in educational institutions, leaving educators underprepared to effectively implement and manage AI tools in the classroom [26]. These issues underscore the necessity of a thoughtful and ethically grounded approach to AI adoption that not only embraces innovation but also safeguards equity, privacy, and the human dimensions of learning.

Despite such ethical and institutional gaps, there is a growing integration of AI tools in classrooms [27]. Their role in teacher education persists and evolves continually [28]. While extensive research has documented the transformative role of AI in education [1, 7], a significant gap remains in empirical studies which explore the dynamic changes in pre-service teachers’ psychological dispositions, such as attitudes, anxiety, acceptance, and innovativeness. Existing studies focusing on pre-service teachers tend to rely on one-time surveys or interviews to examine these constructs in isolation [29–33], leaving open the question of how these intertwined factors jointly enable or inhibit AI uptake among pre-service teachers [34]. In other words, these studies typically track a single outcome and rarely examine the simultaneous evolution of the psychological precursors deemed essential for technology adoption according to the Diffusion of Innovation Theory [35] and the Unified Theory of Acceptance and Use of Technology [36].

AI has transitioned from experimental novelty to mainstream educational practice, prompting ministries of education in countries such as China and the United States to mandate AI literacy across all school levels and teacher education programs [37, 38]. However, no classroom-based intervention has tracked how all four evolve together inside an authentic project-preparation course [39, 40]. Existing intervention research with pre-service teachers suggests that guided or course-embedded use of AI can enhance specific competencies, including pedagogical competence and critical awareness of AI tool deployment [41]. To use AI tools effectively, pre-service teachers must not only understand their functional affordances and limitations but also apply appropriate pedagogical strategies aligned with disciplinary learning goals [42].

This study holds the view that the rapid integration of AI technologies into education presents both opportunities and challenges for teacher education programs. Pre-service teachers, as prospective educators, should be equipped with the skills, confidence, and a positive mindset to effectively leverage AI tools and enhance teaching and learning. However, their readiness to adopt AI is also influenced by psychological factors (e.g., openness to innovation and anxiety) and behavioral intentions (e.g., acceptance and attitudes). In this context, the *Project Preparation in Education* course was selected because it offers a natural setting for hands-on engagement with AI tools across multiple phases of educational research, from problem formulation to literature review and reporting. Its open-ended, inquiry-based design allows pre-service teachers to actively explore the affordances and limitations of AI while developing their research projects, making it a pedagogically relevant space to foster both technical competence and psychological readiness for AI use in teaching. The primary objective of this study is to investigate how participation in an AI-assisted project preparation course affects pre-service teachers’ readiness to apply AI in educational contexts, focusing on key psychological and behavioral variables. Specifically, the study investigates the following questions:

- Are there significant differences between pre-service teachers’ pre-test and post-test scores for individual innovativeness skills after taking the AI-assisted *Project Preparation in Education* course?
- Are there significant differences between pre-service teachers’ pre-test and post-test scores for AI anxiety levels after taking the AI-assisted *Project Preparation in Education* course?
- Are there significant differences between pre-service teachers’ pre-test and post-test scores for AI attitudes after taking the AI-assisted *Project Preparation in Education* course?
- Are there significant differences between pre-service teachers’ pre-test and post-test scores for AI Acceptance after taking the AI-assisted *Project Preparation in Education* course?

### Theoretical framework: psychological antecedents of AI adoption

Grounded in the Diffusion of Innovation theory [35] and the Unified Theory of Acceptance and Use of Technology model [43], this study follows a four-construct lens, namely individual innovativeness, AI anxiety, AI attitudes, and AI acceptance, because together they capture the enabling and inhibiting forces that determine whether pre-service teachers adopt AI tools. These constructs holistically reflect the interplay between personal predispositions, emotional responses, evaluative perceptions, and behavioral intentions.

### Individual innovativeness

Individual innovativeness, defined as a person’s dispositional willingness to try out novel ideas and technologies before others [35], is a pivotal antecedent of technology acceptance among teachers. Large-sample studies on Turkish pre-service teachers have shown that innovativeness predicts higher scores on national technology standards and stronger ICT skills [44, 45]. In AI contexts, personal innovativeness significantly raises behavioral intention to use chatbots for learning [46] and mediates teachers’ adoption of AI research tools [47]. In further intervention studies on pre-service teachers, some reported non-significant changes in individual innovativeness in an educational technology course [48], while others reported significant improvements following online entrepreneurial project training [49], a web-based blended learning programming course [50], and close reading strategies [51]. Moreover, individual innovativeness is associated with their techno-pedagogical education competencies [52] and entrepreneurship skills [53]. A strong positive correlation exists between individual innovativeness and 21st-century skills among nursing students [54]. Additionally, AI has enhanced students’ entrepreneurial competencies, including creativity, opportunity recognition, and idea valuation [55]. However, some students reported experiencing anxiety, fearing that their creativity would be overshadowed by advanced AI technologies [56].

### AI anxiety

AI anxiety in education is an emerging concern as AI becomes more prevalent in learning environments. Recent research with pre-service teachers has revealed specific concerns about AI, with anxiety levels serving as reliable predictors of technology adoption behaviors [57]. In one of these studies, AI anxiety has been found to significantly predict pre-service teachers’ behavioral intentions to design AI-assisted teaching [58]. Furthermore, research has shown that AI learning anxiety and AI job replacement anxiety affect both intrinsic and extrinsic learning motivations, subsequently influencing learning intentions, highlighting the multifaceted nature of AI-related anxiety in educational contexts [59]. In addition, research indicates moderate to high levels of AI anxiety among students and teachers, particularly concerning job displacement and academic integrity [60, 61]. Age, ethnicity, academic achievement, and prior exposure to software education are some of the factors that influence AI anxiety [62, 63]. Studies suggest that integrating AI education into curricula and providing hands-on experiences to students can help reduce anxiety and improve AI literacy [22, 64]. To address AI-related concerns, researchers recommend implementing AI literacy programs in teacher education curricula and providing comprehensive AI education to students [33, 65].

### AI attitude

Attitude is a psychological construct that impacts individuals’ daily decision-making processes and goes beyond their assumptions or ideas. Eagly and Chaiken [66] defined attitudes as “a psychological tendency that is expressed by evaluating a particular entity with some degree of favor or disfavor” (p. 1). Research has demonstrated that attitudes toward AI serve as a sequential mediator in the relationship between AI acceptance and AI anxiety, with AI literacy playing a complementary role [67]. Studies have also identified that teacher acceptance of AI includes components such as perceived ease of use, perceived usefulness, attitude toward AI, and self-efficacy, with attitudes serving as a central organizing construct [29]. Extended technology acceptance models have incorporated attitudes as a key factor in examining teachers’ behavioral intention to use AI tools for teaching and learning, establishing the variable’s theoretical relevance and empirical significance in predicting technology adoption behaviors [68]. 

University students’ attitudes towards AI are multidimensional, comprising cognitive, behavioral, and emotional components [30]. It is reported that students generally have a positive attitude towards the use of AI technologies in higher education [69]. Perceived usefulness, ease of use, and self-efficacy are shown as critical factors affecting pre-service teachers’ intention to utilize AI [29, 70]. Additionally, attitudes, anxiety, and readiness significantly contribute to engagement with AI learning [71, 72]. Pre-service STEM teachers typically exhibit more positive attitudes and lower anxiety toward AI than their non-STEM counterparts [31]. Nevertheless, the overall adoption of AI among pre-service teachers remains low, underscoring a disparity between recognizing AI’s potential and its actual application [73]. Technological Pedagogical Content Knowledge (TPACK) and self-efficacy impact the willingness to integrate AI into STEM education [70]. Socio-cultural factors, such as gender and AI-related experiences, influence students’ attitudes towards AI [74]. AI literacy promotes positive acceptance, while anxiety has a minimal adverse effect [75]. Teachers’ digital competence correlates with favorable attitudes toward AI in educational settings [76]. Therefore, addressing such factors as AI literacy, digital competence, and self-efficacy are crucial for fostering positive attitudes and promoting AI adoption among pre-service teachers.

### AI acceptance

AI acceptance serves as the primary behavioral outcome variable, representing the culmination of various psychological and contextual factors that influence pre-service teachers’ intentions to integrate AI technologies into their educational practice. Research has established that pre-service teachers’ attitudes toward educational technology using AI can affect the learning outcomes of their future students, making acceptance a critical variable for investigation [77]. The Technology Acceptance Model (TAM) provides the theoretical foundation for this variable, and studies demonstrate its effectiveness in predicting factors that may positively or negatively influence behavioral intentions to use AI applications in education [29]. Meta-analytical research has confirmed the robustness of technology acceptance frameworks in predicting AI adoption in educational settings, supporting the inclusion of acceptance as a central outcome variable [78]. A review study on the current state of AI acceptance shows that several key factors influence AI acceptance across various dimensions: technological (i.e., infrastructure readiness, system efficiency, data processing, and personalization), ethical (i.e., data privacy, security risks, algorithmic bias, and transparency), economic (i.e., job displacement concerns and implementation costs), and social (i.e., trust, regulatory compliance, and digital literacy) [79]. Similarly, Zhang et al. [29] found that perceived ease of use and perceived usefulness are primary factors influencing pre-service teachers’ intention to use AI, with gender differences in AI anxiety and perceived enjoyment. Additionally, AI interventions have demonstrated promise in enhancing pre-service teachers’ intentions to use AI-based educational applications [29]. Furthermore, interventions that highlight AI’s usefulness in teaching and learning have proven effective in improving pre-service teachers’ acceptance of AI [80]. Overall, the literature provides a conceptually detailed yet methodologically consistent perspective on pre-service teachers’ innovativeness, anxiety, attitudes, and acceptance of AI. However, its reliance on single-occasion self-report surveys, often conducted outside real AI-supported research project settings, reduces its ecological validity and the depth of its conclusions.

### The project preparation in education

The *Project Preparation in Education* course is a senior undergraduate elective designed to build research literacy and entry-level project design skills. Consistent with national implementations, it is configured as 2–0 (theory–practice) hours per week, two local credits, and 4 ECTS. Its purpose is to develop foundational knowledge of project work in education and to cultivate the introductory skills needed to prepare, execute, and report an educational project. The content covers the project concept and types, scientific research method and its stages, and the preparation of a full project proposal through to reporting and presentation. In this study, the course also integrates guided, ethical use of AI tools to support idea generation, literature synthesis, methodological planning, data analysis, and manuscript drafting, while emphasizing verification of AI outputs, proper attribution, and preservation of students’ academic voice.

## Methods

### Study design

This quasi-experimental study employed a single-group, pre-test and post-test design to explore the research questions [81]. This design involves assessing a dependent variable within a single cohort of participants, both before and after the introduction of a specific intervention. A primary limitation of this approach is its lack of a control or comparison group, which makes it prone to numerous threats to internal validity. Consequently, establishing a definitive causal link between the intervention and the outcome is difficult. The reason for choosing a single-group design was that the sample size was insufficient to divide participants into two groups [82]. In other words, we selected a one‑group pretest–posttest design, because course‑level constraints precluded randomization or a concurrent comparison section. First, the course is offered once per term to a single, capstone‑stage cohort, making parallel sections infeasible. Second, assigning some final‑semester students to a “no‑AI” condition would remove access to tools now widely used in academic writing and research support, which raised ethical and equity concerns. Third, running the intervention in a single cohort minimized cross-sectional contamination (students routinely collaborate across sections), a common threat in departmental settings. Considering these constraints, we treat the pre–post contrast as associational evidence of change rather than a definitive causal effect.

**Fig. 1:**
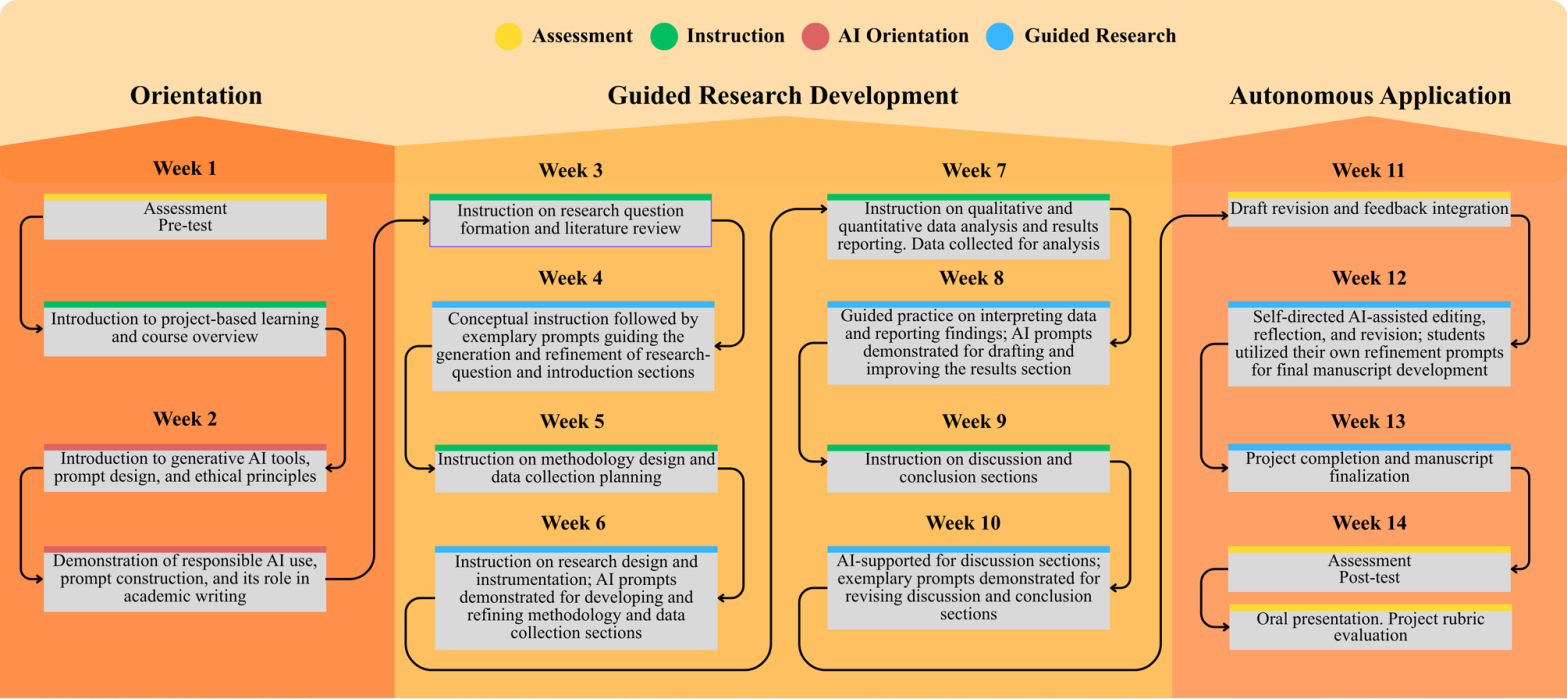
Project Preparation in Education

Figure 1 shows the research design process of this study, highlighting the step-by-step stages from the pre-test to the post-test. It demonstrates how AI tools and instructional components are integrated into project development, covering key phases such as research question development, methodology, data collection, and analysis over 14 weeks.

Figure 1 illustrates the sequential structure of the 14-week intervention, highlighting AI integration and assessment points. The process begins with a pre-test, followed by an introduction to project types in the first week and AI tools in the second week. From the third to the tenth week, a core instructional phase occurs, during which participants receive guidance on the key stages of scientific research—from formulating a research question to data reporting—while concurrently engaging in research development. A critical component of this process, beginning in week two and continuing until the study’s conclusion, is the continuous integration of AI tools to support each stage of research. In the final phase, from weeks 11 to 14, direct instruction is replaced by an autonomous learning approach where students work independently on their projects with instructor feedback. The study concludes after 14 weeks with a post-test to measure the intervention’s effects.

### Participants

In this study, thirty-four fourth-year pre-service teachers (18 women, 16 men; age = 19–29 years) were recruited from a public university located in Türkiye’s Central Anatolia Region. A sensitivity power analysis was conducted using the G*Power software package [83]. The analysis indicated that the study was sufficiently powered (80%) to detect medium to large effects (approximately *d* ≥ 0.50) in the paired-sample comparisons between pre-test and post-test scores [84]. However, the sample size was not sensitive enough to reliably detect small effects (e.g., *d* = 0.20–0.30). Therefore, any non-significant findings should be interpreted with caution, as they may reflect limited statistical power rather than the absence of an actual effect.

Teachers’ participation took place within a vocational elective course offered during the Fall semester of the 2024–2025 academic year. Because the course was open only to students in their final semester, all enrollees during this period constituted the study sample. Participants were distributed across seven educational departments, as presented in Table 1. Accordingly, non-random convenience sampling was employed, whereby participants were selected based on accessibility and availability rather than random assignment [85]. Although this approach expedites data collection and reduces cost, it can limit representativeness and introduce bias, thereby constraining the generalizability and credibility of the findings [86].

**Table 1:** Characteristics of demographic variables

Variable	Category	Frequency	Percentage (%)
Gender	Female	18	52.9%
	Male	16	47.1%
Age	19	3	8.8%
	20	5	14.7%
	21	7	20.6%
	22	7	20.6%
	23	7	20.6%
	24	3	8.8%
	27	1	2.9%
	29	1	2.9%
Department	Elementary Mathematics Education	8	23.5%
	Psychological Counselling and Guidance	6	17.6%
	Science Education	6	17.6%
	Special Education Teaching	5	14.7%
	Elementary School Teacher Education	4	11.8%
	Turkish Language Education	4	11.8%
	Social Studies Education	1	2.9%

Regarding the participants’ technology use and AI experience, as presented in Table 2, the majority reported using both AI and general technology at an intermediate level. Specifically, 70.6% reported intermediate-level AI use, followed by 23.5% at the beginner level and 5.9% at the advanced level. Similarly, 79.4% described their general technology usage as intermediate, while 11.8% identified as advanced users and 8.8% as beginners. These results suggest that, while participants generally demonstrate moderate competence with everyday technologies, their familiarity with AI tools remains comparatively limited.

Regarding technology ownership, 50.0% of participants reported owning both a computer and a smartphone. Other common combinations included a laptop, tablet, and smartphone (20.6%) and a computer, smartphone, and smartwatch (17.6%). A smaller proportion of participants reported owning fewer or more varied combinations of digital devices, indicating some variation in access to technology across the sample.

**Table 2:** Technology use and AI experience

Variable	Category	Frequency	Percentage (%)
Technology Ownership	Computer	1	2.9%
	Computer, Smartphone	17	50.0%
	Tablet, Smartphone	1	2.9%
	Computer, Tablet, Smartphone	7	20.6%
	Computer, Smartphone, Smartwatch	6	17.6%
	Computer, Tablet, Smartphone, Smartwatch	2	5.9%
Technology Use Level	Beginner	3	8.8%
	Intermediate	27	79.4%
	Advanced	4	11.8%
AI Use Level	Beginner	8	23.5%
	Intermediate	24	70.6%
	Advanced	2	5.9%
Daily AI Use During Project	Less than 1 h	5	14.7%
	1–2 h	18	52.9%
	2–3 h	4	11.8%
	3–4 h	4	11.8%
	More than 4 h	3	8.8%

Regarding the participants' daily use of AI tools during the project process, more than half of the participants (52.9%) reported using AI for 1 - 2 hours per day. Additionally, 14.7% used AI for less than one hour, while 11.8% reported daily usage of 2 - 3 hours, and another 11.8% reported usage of 3 - 4 hours. A smaller group (8.8%) reported using AI for more than four hours daily. These findings indicate that although most participants used AI regularly throughout the project, the intensity and duration of use varied among participants.

### Measurement tools

The Individual Innovativeness Scale was developed by Hurt et al. [87] to measure willingness to experience new things and was adapted for Turkish by Kılıçer and Odabaşı [88]. The scale comprises 20 items rated on a 5-point Likert scale (1 = *Strongly Disagree* to 5 = *Strongly Agree*). It includes four subdimensions: resistance to change (RC), opinion-leading (OL), risk-taking (RT), and openness to experience (OE). Items 4, 6, 7, 10, 13, 15, 17, and 20 were reverse-coded so that higher scores indicated greater innovativeness. Mean subdimension scores were calculated by averaging items within each subdimension, and a total innovativeness score was calculated as the mean of all 20 items, where higher values denoted greater individual innovativeness. The reliability coefficients for the subdimensions were as follows: “RC” α = 0.81, “OL” α = 0.73, “OE” α = 0.77, and finally “RT” α = 0.62. Results from an exploratory factor analysis (EFA) supported the four-factor structure, with the factors jointly accounting for 52.521% of the total variance. Specifically, the first factor explained 16.67% of the variance, the second factor 13.61%, the third factor 12.97%, and the fourth factor 9.28%.

The Artificial Intelligence Anxiety Scale (AIAS) was developed by Wang and Wang [89] to measure AI anxiety and was adapted for Turkish by Terzi [90]. The scale comprises 21 items rated on a 7-point scale (1 = *Never* to 7 = *Always*). It consists of four subdimensions: learning (LE), job replacement (JR), sociotechnical blindness (SB), and AI configuration (AIC). Mean subdimension scores were calculated by averaging items within each dimension, and a total anxiety score was computed as the mean of all 21 items, where higher values denoted greater AI-related anxiety. The internal consistency coefficients for each subdimension were: LE = 0.97, JR = 0.92, SB = 0.92, and AIC = 0.96, indicating strong reliability and acceptable internal consistency across all subdimensions. The confirmatory factor analysis results showed that the four-factor structure provided an adequate fit to the data, with TLI = 0.93, CFI = 0.94, SRMR = 0.069, and RMSEA = 0.084.

The Student Attitudes Toward AI Scale was developed by Suh and Ahn [91] to measure student attitudes toward AI and was adapted for Turkish by Derinalp and Ozyurt [92]. The scale consists of 26 items scored on a 5-point Likert scale (1 = *Strongly Disagree* to 5 = *Strongly Agree*). It has three dimensions: behavioral, affective, and cognitive. The behavioral dimension examines students’ observable actions and engagement with AI tools, the affective dimension captures their emotional responses and attitudes toward AI, and the cognitive dimension evaluates their understanding and knowledge of AI concepts. Mean subdimension scores were calculated by averaging items within each dimension, and a total attitude score was computed as the mean of all 26 items, where higher values denoted more positive attitudes toward AI. Internal consistency analyses indicated excellent reliability across the subdimensions, with Cronbach’s alpha values of 0.91 for cognitive, 0.92 for affective, and 0.94 for behavioral. The confirmatory factor analysis results showed goodness of fit to the data, with RMSEA of 0.075, SRMR of 0.047, TLI of 0.93, and CFI of 0.94.

The Generative AI Acceptance Scale was developed by Yilmaz et al. [93] to assess students’ acceptance of AI applications. The scale includes 20 items rated on a 5-point Likert scale (1 = *Strongly Disagree* to 5 = *Strongly Agree*). It has four dimensions: performance expectancy (PE), effort expectancy (EE), facilitating conditions (FC), and social influence (SI). Mean subdimension scores were calculated by averaging items within each dimension, and a total acceptance score was computed as the mean of all 20 items, where higher values denoted greater acceptance of generative AI. Internal consistency analyses indicated excellent reliability across the subdimensions, with Cronbach’s alpha values of 0.96 for PE, 0.96 for SI, 0.96 for FC, and 0.96 for EE. The confirmatory factor analysis indicated an acceptable to good model fit, with CFI = 0.97, GFI = 0.88, IFI = 0.97, TLI = 0.97, RMSEA = 0.067, and SRMR = 0.033.

### Measurement model and conceptual distinctions

Guided by TAM/UTAUT and attitude theory, we conceptualize attitudes toward AI as an evaluative disposition encompassing cognitive, affective, and behavioral assessments of AI. Meanwhile, AI acceptance reflects behavioral intention and enabling beliefs such as performance and effort expectancy, social influence, and facilitating conditions. Both constructs are retained because attitudes can improve independently of intentions if normative pressures or infrastructural beliefs (like facilitating conditions) are weak. Conversely, intention can be high even when emotional responses are ambivalent.

### Data analysis

The study was not pre-registered; therefore, all statistical analyses are presented as exploratory. We controlled family-wise error across the paired comparisons using the Holm–Bonferroni step-down procedure (α = 0.05), reporting both uncorrected and Holm-adjusted *p*-values. Findings should be interpreted cautiously pending confirmatory replication.

Quantitative data were collected via paper-based instruments and analyzed using IBM SPSS Statistics version 28. Before the primary analyses, the dataset underwent a thorough preliminary screening to detect potential outliers, missing values, and violations of normality assumptions. Across all instruments, no missing data or outliers were identified during data screening. Therefore, all analyses were performed on complete cases (*N* = 34). All total and subdimension scores were calculated as mean values, preserving the original response scale metrics (1–5 or 1–7) to maintain interpretability and comparability across constructs.

All variables were measured at the individual level, as each participant completed the scales independently. Although some students collaborated in dyads during project work, all psychological measures were self-reported and analyzed individually. Given the limited number of dyads and the dispositional nature of the constructs, clustering effects were negligible and did not bias the results.

Normality assumptions were evaluated for all five constructs using the Shapiro–Wilk test and descriptive measures (such as skewness and kurtosis). The Shapiro–Wilk test is widely used for assessing normality, particularly for small to moderate sample sizes [94]. As shown in Table 3, except for AI attitude scores, the test results indicated no significant departures from normality for all other construct scores in both the pre-test and post-test. In contrast, AI attitude scores showed statistically significant departures from normality at both measurement points, indicating a non-normal distribution. Nonetheless, when considering the distributions’ shapes via skewness and kurtosis values, all constructs—including attitudes—fell within the generally accepted range of ± 2 [95], supporting the assumption of approximate normality.

Although the Shapiro–Wilk test indicated significant deviations from normality for the attitude variable, the corresponding skewness and kurtosis statistics remained within acceptable limits. Consistent with the recommendations of George and Mallery [95] and Kim [96], parametric tests were considered appropriate for the analyses, given the moderate sample size (*N* = 34) and the approximately normal distribution of the data.

**Table 3:** Shapiro–Wilk test results and distribution characteristics (skewness and kurtosis) for pre-test and post-test scores across all constructs

Construct	Test Type	Pre-Test Score	Post-Test Score
Acceptance	Shapiro–Wilk *p*	0.180	0.216
	Skewness	–0.559	0.032
	Kurtosis	0.733	–0.310
Attitudes	Shapiro–Wilk *p*	0.007*	0.046*
	Skewness	–0.978	–0.954
	Kurtosis	0.614	0.992
Anxiety	Shapiro–Wilk *p*	0.710	0.604
	Skewness	0.337	–0.149
	Kurtosis	–0.011	0.057
Innovativeness	Shapiro–Wilk *p*	0.180	0.216
	Skewness	0.297	0.028
	Kurtosis	0.116	–0.192

*N* = 34 for all constructs. *p* < 0.05*; Shapiro–Wilk test *p*-values assess the normality of score distributions; skewness and kurtosis values further describe the shape and symmetry of the distributions. Values within ± 2 are generally considered acceptable for normality assumptions

A paired-sample t-test was then conducted to compare the measurement scales at two distinct time points: pre-test and post-test. Initially, the analysis assessed whether there were significant differences in the total scores of the measures between the pre-test and post-test. Subsequent data analyses were conducted for each measure to explore changes across its subdimensions. The subdimension-level analysis yielded detailed insights into specific areas of significant change.

To assess the robustness of pre–post differences, Wilcoxon signed-rank tests were conducted as nonparametric alternatives to the paired *t*-tests. Additionally, Pearson correlations between pre- and post-test scores were calculated to evaluate response stability over time.

A significance level of 0.05 was adopted to determine statistical significance. To control for inflation of the familywise error rate across the four comparisons, the Holm–Bonferroni sequential correction was applied [97]. Effect sizes were calculated using Hedges’ *g*_*av*_ with 95% confidence intervals (CI) [98]. Effect sizes were interpreted as trivial (*g*_*av*_ < 0.2), small (*g*_*av*_ ≥ 0.2), medium (*g*_*av*_ ≥ 0.5), or large (*g*_*av*_ ≥ 0.8) [99].

Internal consistency reliabilities were assessed for all constructs at both pre- and post-test. Cronbach’s α values for AI acceptance (pre: 0.93, post: 0.95), AI anxiety (pre: 0.95, post: 0.95), and AI attitude (pre: 0.97, post: 0.96) indicate excellent internal consistency and temporal stability. In contrast, Individual innovativeness showed poor reliability (pre: 0.36, post: 0.31), suggesting measurement issues that limit the interpretability of observed changes for this construct.

### The context of the study

A 14-week *Project Preparation in Education* course was delivered during the fall semester to enhance pre-service teachers’ research literacy skills. Before data collection began, all teachers received a detailed briefing on the study’s aims and procedures and provided written informed consent, affirming their voluntary participation.

Before using any AI platform, students were informed about the ethical use of AI during their research preparation. During the session, it was emphasized that AI could make mistakes and that AI-generated outputs were needed to be double-checked, just as human-generated information does. Specifically, four rules that students should follow whenever they use an AI tool were outlined: (1) check for bias and errors by cross-verifying facts before accepting suggestions; (2) use AI as an assistant, not a crutch, by keeping original thinking at the core of their work; (3) respect copyright by citing sources the AI references, paraphrasing instead of copying, and never reusing protected text without permission; and (4) maintain a human voice by revising AI output to match their disciplinary style and tone.

Table 4 outlines the course structure and core concepts covered. The main goal was to equip participants with the skills to conduct AI-assisted scientific research and write articles. AI models, such as ChatGPT Plus, Elicit, Consensus, Gemini, NotebookLM, and Perplexity, were used as supportive tools to aid teachers in writing, refining, and editing the key sections of their scientific projects, including research questions, introduction, literature review, methods, results, discussion, and conclusion.

Specifically, the first week was dedicated to familiarizing students with the concept of projects and various project programs implemented in educational settings. The second week introduced AI tools, focusing on writing effective prompts to enhance academic writing, perform data analysis, and present reports. Over the subsequent seven weeks, the course covered distinct stages of scientific project development, demonstrating how to leverage AI tools to facilitate each phase.

During the third week, students received instruction on developing research questions for both qualitative and quantitative studies. They initiated the formulation of their questions by using AI tools to generate and refine them based on their research interests. In the following stages, the same pedagogical strategy guided students through each phase of the AI-assisted research process.

During the final five weeks (weeks 10–14), the instructional approach transitioned from direct teaching to coaching. Students worked independently on developing full-length research manuscripts, using peer-reviewed examples as benchmarks and iteratively improving their AI-generated drafts. The instructor provided formative feedback, emphasizing both the ethical use of AI and the development of disciplinary rigor. A strong focus was placed on integrating authentic data and fostering recursive human–AI collaboration, ensuring that AI tools were positioned as aids rather than replacements for critical academic skills.

By the end of the semester, participants, either individually or in groups, had successfully produced a complete manuscript aligned with APA guidelines and had acquired substantial practical experience in ethically and effectively employing AI tools throughout the entire research process. This experience fostered the development of applied research literacy and the translation of theoretical understanding into actionable competencies.

It should be noted that, since these students had previously completed the *Measurement and Evaluation* and *Scientific Research Methods* courses in their fourth semester, they had a foundational understanding of formulating research questions, selecting appropriate research designs, and interpreting empirical data. However, their research skills remained limited due to insufficient opportunities to apply theoretical knowledge in authentic research contexts, minimal exposure to data analysis techniques, and a lack of sustained mentorship in designing and conducting independent empirical studies.

**Table 4:** Project Preparation in Education: course outline and key concepts

Week	Subject	Description
1	Project Concept and Project Types	Introduction to project-based learning, defining project concepts, exploring various project types (scientific, educational, social, etc.), and understanding their significance in education.
	Project Programs in Schools	Overview of national and international project programs implemented in schools (e.g., Erasmus+, TÜBİTAK), their objectives, and application processes.
2	AI Tools	Exploring the use of AI tools, its capabilities in idea generation, literature summarization, academic writing enhancement, and data analysis support, as well as effective prompt-writing strategies and ethical considerations for responsible AI usage in educational research.
	Stages of a Scientific Project	Examination of the fundamental steps in conducting a scientific project, including problem identification, planning, execution, and dissemination of results.
3	Research Question*	Developing a clear and focused research question, understanding its role in guiding a study, and distinguishing between different types of research questions. Note: The beginning of project development
4	Introduction*	Writing an effective introduction, stating the research problem, defining objectives, and explaining the significance of the study.
5	Literature Review*	Conducting a comprehensive review of existing literature, identifying gaps in research, and synthesizing relevant studies to build a strong foundation for the project.
6	Research Methodology*	Understanding different research designs (qualitative, quantitative, mixed methods), selecting appropriate methodologies, and justifying methodological choices.
7	Data Collection*	Exploring data collection techniques such as surveys, interviews, observations, and experiments, while ensuring reliability and validity.
8	Data Analysis*	Introduction to qualitative and quantitative data analysis techniques, using statistical tools, and interpreting results.
9	Data Reporting*	Structuring a research report, presenting findings effectively, and discussing implications for practice and further research.
10–14	Project Development	Guiding students through the iterative process of refining their research projects, incorporating feedback, and finalizing their studies for presentation or publication.

*AI Tools integrated

The project groups in this study consisted of one or two members, depending on individual preferences. There were 24 total project groups, with 10 having two members and 14 having one member. Grading AI-assisted scientific research projects required a structured evaluation framework to ensure consistency and fairness in the assessment process. Since no existing measurement or evaluation tool was available for assessing student-developed projects, a rubric was adapted from previously validated and reliable scales [100, 101]. This rubric was used to evaluate projects across six sections: introduction, literature review, methodology, results, discussion, and conclusion. The course instructor graded all the projects on a scale of 0 to 100 across six sections, using an adapted rubric.

The adapted rubric was used solely for instructional feedback and grading. We did not analyze rubric scores, because (a) the study focused on psychological outcomes (i.e., innovativeness, anxiety, attitudes, acceptance), and (b) conflating product scores with these constructs risks criterion contamination (i.e., product quality can reflect prior design skill or team dynamics rather than shifts in dispositions). We therefore report the rubric for transparency but exclude its scores from statistical analyses.

## Results

This section delineates the findings for each research question. The results include both the overall construct and its subdimensions, providing a comprehensive analysis of the data. Descriptive statistics and pertinent tables underscore the key differences identified in the data, especially concerning the paired-samples t-test.

### Preliminary analyses

Table 5 presents the intercorrelations among the four primary constructs at both measurement points. As predicted, AI attitudes and AI acceptance were strongly and positively correlated, whereas AI anxiety was negatively associated with both constructs. Individual innovativeness correlated weakly and positively with acceptance and attitudes at pre-test, and these associations became nonsignificant after the intervention. All intercorrelations were below 0.85, supporting adequate discriminant validity and the conceptual distinctiveness between attitudes and acceptance [102]. Full correlation matrices for all subdimensions are provided in the supplementary materials.

**Table 5:** Pearson correlations among the four main constructs at pre-test and post-test

Variable	1	2	3	4
Pre-test				
1. AI Acceptance	-			
2. AI Attitudes	0.84***	-		
3. AI Anxiety	−0.47**	−0.55***	-	
4. Individual Innovativeness	0.26	0.32	−0.30	-
Post-test				
1. AI Acceptance	-			
2. AI Attitudes	0.78***	-		
3. AI Anxiety	−0.54***	−0.66***	-	
4. Individual Innovativeness	0.11	−0.07	0.19	-

*N* = 34. Values are Pearson’s *r* (two-tailed). *p < 0.05, **p < 0.01, *** *p <* 0.001

### RQ 1. Do pre-service teachers’ innovativeness skills differ between pre-test and post-test?

Based on the data presented in Table 6, the results indicated a statistically significant difference between pre- and post-test scores for only individual innovativeness and opinion leading subdimension.

**Table 6:** Paired-sample t-tests comparing pre-test and post-test scores for individual innovativeness and its subdimensions

Construct	Pre M (SD)	Post M (SD)	t	df	*p*-value	Mean Diff [95% CI]	Hedges’ g_av_ (Interpretation)
Individual innovativeness	3.30 (0.26)	3.41 (0.24)	−2.05	33	0.048*	−0.11 [− 0.23, − 0.00]	0.44 (Small)
Resistance to change	2.74 (0.59)	2.81 (0.62)	−0.70	33	0.487	−0.07 [− 0.29, 0.14]	0.12 (Trivial)
Opinion leading	3.57 (0.65)	3.78 (0.52)	−2.09	33	0.044*	−0.21 [− 0.42, − 0.01]	0.35 (Small)
Openness to experience	3.89 (0.41)	3.98 (0.43)	−1.48	33	0.150	−0.09 [− 0.21, 0.03]	0.21 (Small)
Risk taking	3.37 (0.83)	3.46 (0.79)	−0.54	33	0.595	−0.09 [− 0.42, 0.25]	0.11 (Trivial)

*N* = 34. *p*-values are two-tailed; *p < 0.05

According to the results, the individual innovativeness increased from pre-test (*M* = 3.30, *SD* = 0.26) to post-test (*M* = 3.41, *SD* = 0.24), *t* (33) = − 2.05, *p* = 0.048, with a small effect size (Hedges’ *g*_*av*_ = − 0.34). Similarly, the opinion leading subdimension showed a significant increase, *t* (33) = − 2.09, *p* = 0.044, with a small effect size (Hedges’ *g*_*av*_ = − 0.35), indicating that participants perceived themselves as slightly more likely to influence others regarding innovative practices. However, no significant differences were found in the resistance to change subdimension, *t* (33) = − 0.70, *p* = 0.487, openness to experience, *t* (33) = − 1.48, *p* = 0.150, or risk taking, *t* (33) = − 0.54, *p* = 0.595. The effect sizes for these non-significant dimensions were trivial or small (Hedges’ *g*_*av*_ ≤ 0.25), suggesting minimal changes.

Paired-samples correlations were conducted to examine the relationships between pre-test and post-test scores for individual innovativeness and its subdimensions. The results showed a weak, non-significant correlation for the individual innovativeness (*r* = 0.18, *p* = 0.32) and the risk taking subdimension (*r* = 0.30, *p* = 0.08). In contrast, significant positive correlations were found for the resistance to change subdimension (*r* = 0.49, *p* = 0.003), the opinion leading subdimension (*r* = 0.51, *p* = 0.002), and the openness to experience subdimension (*r* = 0.66, *p* < 0.001), indicating moderate to strong consistency between pre-test and post-test scores on these subdimensions.

After applying the Holm–Bonferroni sequential correction for multiple comparisons, none of the paired-sample differences remained statistically significant. Specifically, non-significant improvements were observed from pre-test to post-test in the individual innovativeness subdimension (*p* = 0.048, corrected *p* = 0.240) and in the opinion leadership subdimension (*p* = 0.044, corrected *p* = 0.220), indicating that the initially significant results should be interpreted with caution. Although mean scores demonstrated slight increases across all variables, the corrected analyses suggest that the AI-intervention in the course produced only small and statistically non-robust changes in participants’ innovativeness tendencies.

Wilcoxon signed-rank tests were conducted as nonparametric alternatives to the paired t-tests. According to the results, no significant differences were found for individual innovativeness (*Z* = 1.86, *p* = 0.062) and its three subdimensions: resistance to change (*Z* = 0.64, *p* = 0.524), openness to experience (*Z* = 1.48, *p* = 0.139), and risk taking (*Z* = 0.84, *p* = 0.403). However, a significant difference was observed for the opinion leading subdimension (*Z* = 1.99, *p* = 0.047), indicating that participants’ opinion-leading tendencies significantly increased following the intervention. These results suggest that while the intervention did not produce significant changes across most dimensions of individual innovativeness, it effectively enhanced participants’ ability to influence and guide others’ opinions.

### RQ 2. Do pre-service teachers’ levels of AI anxiety change after the course?

Based on the data presented in Table 7, the results indicated no statistically significant differences between pre-test and post-test scores for AI anxiety or any of its subdimensions.

**Table 7:** Paired-sample t-tests comparing pre-test and post-test scores for AI anxiety and its subdimensions

Construct	Pre M (SD)	Post M (SD)	t	df	*p*-value	Mean Diff [95% CI]	Hedges’ g_av_ (Interpretation)
AI Anxiety	3.69 (1.27)	3.74 (1.24)	−0.29	33	0.772	−0.06 [−0.44, 0.33]	0.04 (Trivial)
Job Replacement	4.39 (1.45)	4.32 (1.62)	0.29	33	0.776	0.07 [−0.43, 0.57]	−0.05 (Trivial)
Sociotechnical Blindness	3.87 (1.39)	3.97 (1.37)	−0.43	33	0.672	−0.10 [−0.59, 0.39]	0.07 (Trivial)
AI Configuration	4.01 (1.78)	4.08 (1.54)	−0.22	33	0.830	−0.07 [−0.71, 0.57]	0.04 (Trivial)
Learning	3.96 (1.26)	3.83 (1.29)	0.64	33	0.525	0.13 [−0.28, 0.53]	−0.11 (Trivial)

*N* = 34. *p*-values are two-tailed; *p < 0.05

According to the results, AI anxiety did not change significantly from pre-test (*M* = 3.69, *SD* = 1.27) to post-test (*M* = 3.74, *SD* = 1.24), *t* (33) = − 0.29, *p* = 0.77, with a negligible effect size (Hedges’ *g*_*av*_ = − 0.05). Similarly, no significant differences were observed across the four subdimensions of AI anxiety. The job replacement subdimension showed a slight but non-significant decrease, *t* (33) = 0.29, *p* = 0.78, Hedges’ *g*_*av*_ = 0.05, while the sociotechnical blindness subdimension showed a slight, non-significant increase, *t* (33) = − 0.43, *p* = 0.67, Hedges’ *g*_*av*_ = − 0.07. The AI configuration subdimension remained essentially unchanged, *t* (33) = − 0.22, *p* = 0.83, Hedges’ *g*_*av*_ = − 0.04, and the learning subdimension showed a slight, non-significant decrease, *t* (33) = 0.64, *p* = 0.53, Hedges’ *g*_*av*_ = 0.11.

Paired-samples correlations were conducted to examine the relationships between pre- and post-test scores for AI anxiety and its subdimensions. Moderate to strong positive correlations were observed for AI anxiety (*r* = 0.61, *p* < 0.001) and four subdimensions: job replacement (*r* = 0.57, *p* < 0.001), sociotechnical blindness (*r* = 0.48, *p* = 0.004), AI configuration (*r* = 0.39, *p* = 0.023), and learning (*r* = 0.59, *p* < 0.001). These findings indicate consistent response patterns across time, suggesting that although participants’ overall anxiety toward AI did not change significantly, their relative rankings on each variable remained stable from pre- to post-test.

After applying the Holm–Bonferroni correction for multiple comparisons, all results remained nonsignificant. All observed effect sizes were trivial, indicating that the differences between pre- and post-test scores were minimal. Given the study’s sample size, the analysis was adequately powered to detect medium-to-large effects; therefore, the nonsignificant findings suggest that any potential changes in AI anxiety were likely small in magnitude.

Wilcoxon signed-rank tests were also conducted as nonparametric alternatives to the paired *t*-tests. According to the results, no significant differences were found for AI anxiety (*Z* = − 0.41, *p* = 0.68) and its subdimensions: job replacement (*Z* = − 0.65, *p* = 0.52), sociotechnical blindness (*Z* = 0.11, *p* = 0.91), AI configuration (*Z* = − 0.01, *p* = 0.99), or learning (*Z* = − 0.63, *p* = 0.53). These results corroborate the paired *t*-test findings, indicating that participants’ anxiety toward AI remained stable following the course.

### Do pre-service teachers’ attitudes toward AI differ between pre-test and post-test?

Based on the data presented in Table 8, the paired-samples t-test results showed a significant difference between pre- and post-test scores for AI acceptance and its three subdimensions.

**Table 8:** Paired-sample t-tests comparing pre-test and post-test scores for AI attitudes and its subdimensions

Construct	Pre M (SD)	Post M (SD)	t	df	*p*-value	Mean Diff [95% CI]	Hedges’ g_av_ (Interpretation)
AI Attitudes	3.36 (0.85)	3.83 (0.68)	−3.80	33	< 0.001**	−0.48 [−0.73, −0.22[	−0.64 (Medium)
Cognitive	3.17 (0.78)	3.50 (0.67)	−2.83	33	0.008**	−0.33 [−0.57, −0.09]	−0.47 (Small)
Affective	3.34 (0.91)	3.93 (0.77)	−4.01	33	< 0.001**	−0.59 [−0.90, −0.29]	−0.67 (Medium)
Behavioral	3.43 (0.88)	3.87 (0.67)	−3.34	33	0.002**	−0.43 [−0.69, −0.17]	−0.56 (Medium)

*N* = 34. *p*-values are two-tailed; *p < 0.05, **p < 0.01

The results revealed a significant improvement in AI attitudes from pre-test (*M* = 3.36, *SD* = 0.85) to post-test (*M* = 3.83, *SD* = 0.68), *t*(33) = − 3.80, *p* < 0.001, Hedges’ *g*ₐ_v_ = − 0.64, indicating a medium effect. Significant improvements were also observed across all three subdimensions. The cognitive subdimension increased significantly from pre-test (*M* = 3.17, *SD* = 0.78) to post-test (*M* = 3.50, *SD* = 0.67), *t*(33) = − 2.83, *p* = 0.008, with a small effect size (Hedges’ *g*_*av*_ = − 0.47), reflecting enhanced understanding and beliefs about AI’s potential. The affective subdimension also improved significantly, *t*(33) = − 4.01, *p* < 0.001, with a medium effect (Hedges’ *g*_*av*_ = − 0.67), suggesting more positive emotional responses toward AI. Additionally, the behavioral subdimension increased significantly, *t*(33) = − 3.34, *p* = 0.002, with a medium effect (Hedges’ *g*_*av*_ = − 0.56), indicating greater willingness to engage with or use AI technologies.

Paired-samples correlations were conducted to examine the relationships between pre- and post-test scores for AI attitude and its subdimensions. Moderate to strong positive correlations were observed for the AI attitudes (*r* = 0.56, *p* < 0.001), the cognitive subdimension (*r* = 0.57, *p* < 0.001), the affective subdimension (*r* = 0.48, *p* = 0.004), and the behavioral subdimension (*r* = 0.56, *p* < 0.001). These findings indicate consistent response patterns across time, suggesting that while participants’ mean scores improved, their relative rankings remained stable.

After applying the Holm–Bonferroni correction for multiple comparisons, all paired-sample t-tests remained statistically significant. Thus, significant improvements were observed from pre-test to post-test in AI attitudes (*p* < 0.001, corrected *p* = 0.005), cognitive subdimension (*p* = 0.008, corrected *p* = 0.032), affective subdimension (*p* < 0.001, corrected *p* = 0.005), and behavioral subdimension (*p* = 0.002, corrected *p* = 0.010). Thus, all four components of AI attitudes demonstrated significant gains after the intervention, even after adjustment for multiple testing.

Wilcoxon signed-rank tests were also conducted as nonparametric alternatives to the paired *t*-tests. Results revealed statistically significant increases in AI attitudes (*Z* = 3.40, *p* < 0.001), the cognitive subdimension (*Z* = 2.57, *p* = 0.010), the affective subdimension (*Z* = 3.40, *p* < 0.001), and the behavioral subdimension (*Z* = 3.18, *p* = 0.001). These findings corroborate the results of the parametric analyses, underscoring the robustness of the observed pre–post gains in AI attitudes and confirming that the intervention produced meaningful and reliable positive changes across cognitive, affective, and behavioral dimensions.

### Do pre-service teachers’ levels of AI acceptance differ between pre-test and post-test?

Based on the data presented in Table 9, the paired-samples t-test results showed a significant difference between pre- and post-test scores for AI acceptance and its four subdimensions.

**Table 9:** Paired-sample t-tests comparing pre-test and post-test scores on AI acceptance and its subdimensions

Construct	Pre M (SD)	Post M (SD)	t	df	*p*-value	Mean Diff [95% CI]	Hedges’ g_av_ (Interpretation)
AI acceptance	3.51 (0.66)	3.96 (0.57)	−4.020	33	< 0.001**	−0.44 [−0.67, −0.22]	−0.70 (Medium)
Performance expectancy	3.79 (0.68)	4.05 (0.59)	−2.14	33	0.040*	−0.26 [−0.50, −0.01]	−0.40 (Small)
Social influence	3.04 (1.03)	3.79 (0.77)	−4.55	33	< 0.001**	−0.75 [−1.09, −0.42]	−0.82 (Large)
Effort expectancy	3.53 (0.84)	3.89 (0.71)	−2.72	33	0.010*	−0.36 [−0.64, −0.09]	−0.46 (Small)
Facilitating conditions	3.63 (0.77)	4.12 (0.51)	−3.67	33	< 0.001**	−0.49 [−0.76, −0.22]	−0.75 (Medium)

*N* = 34. *p*-values are two-tailed; *p < 0.05, **p < 0.01

According to the results, AI acceptance increased significantly from pre-test (*M* = 3.51, *SD* = 0.66) to post-test (*M* = 3.96, *SD* = 0.57), *t* (33) = −4.02, *p* < 0.001, with a medium effect size (Hedges’ *g*_*av*_ = −0.70). Similarly, the performance expectancy subdimension showed a significant increase, *t*(33) = −2.14, *p* = 0.040, with a small effect size (Hedges’ *g*_*av*_ = −0.40), indicating a greater belief in the utility and effectiveness of AI. The social influence subdimension improved significantly, *t*(33) = −4.55, *p* < 0.001, with a medium effect size (Hedges’ *g*_*av*_ = −0.82), suggesting stronger perceived endorsement of AI use from peers or the academic environment. Significant gains were also found in the effort expectancy subdimension, *t*(33) = −2.72, *p* = 0.010, with a medium effect size (Hedges’ *g*_*av*_ = −0.46), indicating greater perceived ease of use. Finally, the facilitating conditions subdimension increased from pre- to post-test, *t*(33) = −3.67, *p* < 0.001, with a medium effect (Hedges’ *g*_*av*_ = −0.75), reflecting stronger perceptions of support and access to resources.

Paired-samples correlations were conducted to examine the relationships between AI acceptance and its subdimensions. Moderate and statistically significant correlations were observed for AI acceptance (*r* = 0.46, *p* = 0.006) and its three subdimensions: performance expectancy (*r* = 0.39, *p* = 0.021), social expectancy (*r* = 0.46, *p* = 0.007), and effort expectancy (*r* = 0.50, *p* = 0.003), indicating a consistent pattern of responses across the two time points. In contrast, the correlation for facilitating expectancy subdimension was weaker and not significant (*r* = 0.31, *p* = 0.073), suggesting greater variability in responses across time.

After applying the Holm–Bonferroni correction for multiple comparisons, all paired-sample t-tests remained statistically significant. Specifically, significant improvements were observed in participants’ AI acceptance (*p* < 0.001, corrected *p* = 0.005), performance expectancy subdimension (*p* = 0.040, corrected *p* = 0.040), effort expectancy subdimension (*p* = 0.010, corrected *p* = 0.025), social expectancy subdimension (*p* < 0.001, corrected *p* = 0.005), and facilitating expectancy subdimension (*p* < 0.001, corrected *p* = 0.005).

Wilcoxon signed-rank tests were conducted as nonparametric alternatives to the paired *t*-tests to assess the robustness of pre–post differences. Results revealed statistically significant increases in AI acceptance (*Z* = 3.24, *p* = 0.001), the effort expectancy subdimension (*Z* = 3.44, *p* < 0.001), the social expectancy subdimension (*Z* = −3.80, *p* < 0.001), and the facilitating expectancy subdimension (*Z* = 3.25, *p* = 0.001). However, the change in performance expectancy subdimension was not statistically significant (*Z* = 1.81, *p* = 0.071).

### Discussion and conclusion

The present study investigated whether integrating AI tools into a 14-week, project-based research course could meaningfully influence pre-service teachers’ psychological readiness for educational AI adoption. After correcting for multiple comparisons, statistically significant and practically meaningful improvements were observed in AI attitudes and AI acceptance, whereas AI-related anxiety and individual innovativeness remained comparatively stable. Reliability coefficients indicated strong internal consistency across most constructs, supporting the validity of the detected changes. However, caution is warranted in interpreting innovativeness outcomes, as internal consistency for this construct was notably low. These findings indicate that exposure to AI tools can produce statistically significant improvements in specific dimensions of technological attitudes and acceptance, while simultaneously underscoring domains that may require more intensive or prolonged interventions.

This study found a small pre–post improvement in individual innovativeness, particularly in the opinion-leadership subdimension. Although these gains did not remain statistically significant after correcting for multiple comparisons, they offer preliminary evidence that authentic, collaborative projects may foster pre-service teachers’ readiness to advocate for novel practices. This finding aligns with previous research showing that project-based learning activities incorporating peer feedback and public presentation can increase educators’ willingness to promote new ideas [49] and that early adopters often exhibit heightened opinion leadership following design-focused coursework [50]. Our results further extend this literature by suggesting that embedding AI tools within authentic, data-driven projects may enhance pre-service teachers’ confidence in disseminating ideas. This interpretation is supported by Ayvaz Öztürk’s [103] work, which identifies opinion leadership as a strong predictor of digital nativeness among future educators, and by recent studies showing that AI-supported project cycles can strengthen perceived entrepreneurial competencies in higher education contexts [55].

In contrast, other subdimensions of individual innovativeness, namely, resistance to change, risk-taking, and openness to experience, remained statistically stable throughout the intervention. This pattern aligns with longitudinal research on personality and innovation-related traits, which consistently shows that dispositions such as risk propensity and openness develop incrementally over extended periods or in response to significant life transitions [104]. Consistent with this perspective, Weis et al. [105] observed high test–retest reliability in resistance-to-change scores across an academic year, reinforcing the notion that such traits exhibit limited malleability during short-term interventions [106]. In the context of teacher education, Kulaksız [48] similarly reported no significant pre–post improvement in innovativeness following an eight-week educational technology course, attributing this outcome to the limited duration of exposure. However, findings from a 15-week design-thinking module with psychology undergraduates revealed moderate gains in openness to experience, suggesting that the extent of trait plasticity may be moderated by instructional intensity, disciplinary orientation, and cultural context [107]. Taken together, these results indicate that while socially oriented facets of innovativeness such as opinion leadership may respond readily to short-term, collaborative, and AI-supported learning experiences, more deeply ingrained personality-linked dimensions remain relatively stable. This interpretation highlights the importance of sustained, iterative engagement with AI-mediated, project-based learning environments to facilitate broader dispositional growth, as suggested by recent reviews of large language model applications in higher education [107] and by emerging evidence from ChatGPT-assisted lesson-planning studies [108].

The study also demonstrated statistically reliable gains in AI attitudes across the cognitive, affective, and behavioral subdimensions. Comparable multi-component attitude gains have been reported in recent experiential-learning studies with teacher candidates and undergraduates, where repeated interaction with AI tools fostered more favorable beliefs about usefulness, heightened enjoyment, and stronger enactment intentions [109]. Students’ motivational and attitudinal indices improved after an eight-week interactive scaffolding trial, during which they practiced language skills with AI chatbots [110]. A mixed-methods program linking AI literacy with reflective journals also reported attitude shifts attributable to iterative practice and self-evaluation [111]. Similarly, a quasi-experimental study reported a significant change in university students’ positive attitudes toward AI [112]. There are also contradictory findings. A recent exploratory study reported only marginal cognitive-attitude gains after unguided use of ChatGPT, attributing the limited shift to superficial interaction and the absence of instructor mediation [30].

Parallel gains were observed in AI acceptance and its sub-dimensions—performance expectancy, effort expectancy, social influence, and facilitating conditions —aligning with the Extended Technology Acceptance Model and the Unified Theory of Acceptance and Use of Technology. These theories show that perceived usefulness and ease of use (performance and effort expectancy) are decisive predictors of pre-service teachers’ behavioral intention to adopt AI applications [77, 113], while structured, hands-on courses similar to this study strengthen those beliefs and translate them into higher acceptance indices [109]. The pronounced rise in social influence aligns with evidence that peer collaboration, instructor endorsement, and normative pressure significantly increase the willingness to deploy AI tools in educational contexts [114, 115]. Likewise, the improvement in facilitating conditions parallels findings that ready access to AI platforms, technical guidance, and institutional support lowers adoption barriers for pre-service teachers [76, 116]. Taken together, our results reinforce meta-analytic conclusions that multifaceted training environments—those combining experiential practice with robust organizational backing—yield the most significant gains in AI acceptance [117].

In contrast, AI anxiety—including its learning, job-replacement, sociotechnical-blindness, and AI-configuration subdimensions—remained statistically unchanged. This stability aligns with prior research indicating that pre-service teachers’ apprehensions about automation, ethical opacity, and skill atrophy often persist despite short-term exposure to AI applications [33, 39, 60, 61]. Concerns related to job loss, moral dilemmas, and the potential diminishment of human agency can endure even after direct engagement with AI technologies. Regarding the sub-dimensional analyses, worries about automation-driven job loss appear particularly resistant to change in brief interventions, as reflected in the unchanged job-replacement subdimension. Longitudinal survey evidence with both employees and university students similarly demonstrates that job-loss anxiety persists even when participants receive hands-on AI experience or assurances of upskilling support [118, 119]. The persistence of elevated scores on the sociotechnical blindness subdimension further suggests that many participants may continue to perceive AI as an opaque “black box,” whose hidden decision-making processes might yield unpredictable or harmful societal consequences. Consistent findings from classroom studies indicate that teacher candidates and undergraduates often remain skeptical of AI transparency and accountability even after structured instruction [120].

The persistence of anxiety likely reflects the pedagogical design of the present course, which primarily emphasized technical competence and functional application—how to write prompts, check outputs, and complete each manuscript section—rather than affective engagement. The AI intervention effectively equipped participants with practical AI skills related to research, writing, and feedback processes; however, it offered limited opportunities for critical reflection on the emotional, ethical, and sociocultural implications of AI use. In essence, participants learned to use AI effectively but had fewer structured opportunities to reflect on its broader educational and societal ramifications. Prior research on technology integration suggests that skill-focused designs tend to enhance perceptions of usefulness and ease of use but exert minimal influence on deeper affective constructs, such as anxiety, uncertainty, or moral ambivalence [109]. Future interventions would therefore benefit from embedding affect-focused pedagogical strategies that explicitly address the emotional and ethical dimensions of AI adoption. Evidence-informed approaches may involve guided reflective journaling to assist participants in articulating their evolving feelings and value tensions, structured ethical dialogues and case-based simulations to examine issues of autonomy, fairness, and accountability, collaborative peer-mentoring sessions to promote social sense-making, and mindfulness-based reflection activities to enhance emotional awareness resilience. Empirical evidence indicates that combining technical skill development with reflective inquiry and ethical discussion can yield modest yet significant reductions in AI-related anxiety [121].

Theoretical works further substantiate the findings on AI anxiety. Johnson and Verdicchio [122] argued that AI anxiety persisted when public discourse framed intelligent systems as autonomous agents, thereby obscuring continuing human oversight and governance; without explicit examination of such sociotechnical boundaries, users were unlikely to recalibrate their fears. Considering these perspectives and the empirical pattern observed here, it seems plausible that a single 14-week course—no matter how rich in hands-on AI practice—may not be sufficient to dismantle entrenched apprehensions tied to employment security, ethical uncertainty, and perceived loss of human agency. The current study results also support a cautious endorsement of educational AI, alongside recent evidence indicating that AI-assisted instruction can enhance students’ higher-order thinking [123] and problem-solving skills [2]. AI is most effective when it complements rather than replaces learners’ cognitive efforts. Yet, these benefits must be balanced against potential risks. Large-scale surveys and reviews warn that overdependence on AI could weaken critical thinking, promote plagiarism, and reduce students’ resilience [124, 125]. Ultimately, AI’s impact on higher-order thinking depends on instructional quality and proper governance; it can either boost or diminish cognitive skills.

While the course yielded clear improvements in AI attitudes and acceptance, the persistence of anxiety underscores the complex interplay between cognitive-evaluative and affective-moral appraisals. This pattern aligns with the prior observation that well-designed AI training can enhance AI acceptance and foster positive attitudes toward AI among pre-service teachers [109]. This coexistence of positive attitudes and enduring anxieties mirrors prior observations that individuals can hold favorable views of AI’s utility while simultaneously fearing its ethical or occupational consequences [32, 126]. Two factors may account for this pattern. First, the AI intervention primarily emphasized skill acquisition and instrumental integration (e.g., research support, drafting, feedback cycles). Such exposure can improve perceived usefulness and ease of use without directly engaging the affective and moral appraisals that underpin anxiety (e.g., employability, academic integrity, opacity, surveillance). Second, the semester coincided with high-salience public discourse on both the opportunities and risks of AI. Heightened ambient risk signals can stabilize or even elevate anxiety, potentially offsetting any desensitization from classroom practice. Taken together, these dynamics suggest that attitude and acceptance shifts may be achieved through authentic, scaffolded use, whereas meaningfully reducing anxiety likely requires explicit, affect-focused pedagogy that surfaces and processes fears, values tensions, and uncertainty.

In summary, the findings demonstrate that integrating AI tools into an education course encompassing a series of research-based tasks, such as drafting and refining the Abstract, Introduction, Method, Results, and Discussion sections, can meaningfully enhance pre-service teachers’ readiness for AI integration by improving attitudes and acceptance while fostering limited gains in social innovativeness. However, the persistence of AI-related anxiety and the stability of deeper personality traits indicate that affective and dispositional change necessitates longer, reflective, and iterative engagement. For teacher education programs, this suggests that adequate AI preparation must move beyond functional training to encompass ethical reasoning, critical reflection, and emotional support. A holistic approach—one that cultivates technical competence, self-efficacy, and emotional resilience—is essential to empower future educators to harness AI’s transformative potential responsibly and confidently within their classrooms.

These findings should be interpreted as analytic rather than statistical generalizations. The study was conducted with a single cohort of fourth-year pre-service teachers in one 14-week project-based course taught by one instructor, with structured, ethically guided access to AI tools. As such, the most plausible transfer is to programs that share comparable course goals (project preparation in education), instructional design (authentic projects with scaffolded AI prompts), low-to-moderate baseline familiarity with AI, and supportive institutional norms and policies. Caution is warranted when extending conclusions to markedly different contexts (e.g., K–12 students, in-service teachers, courses with restrictive AI policies, or settings with substantially different linguistic/cultural and assessment regimes).

### Implications and future directions

The findings from this study have several important implications for teacher education programs, teaching practices, and policy initiatives focused on preparing educators for AI-driven educational environments. The results show that a structured, project-based course can significantly improve pre-service teachers’ creativity, attitudes toward AI, and acceptance of AI tools. However, the lack of a significant decrease in AI anxiety indicates that short-term, hands-on experience alone may not be enough to ease deeper concerns about automation and job security. This emphasizes the need for a more comprehensive approach to integrating AI in teacher preparation.

A key implication is the need to integrate genuine AI experiences into teacher education curricula. The notable improvements in opinion leadership, attitudes, and acceptance indicate that practical application is essential for cultivating a positive outlook toward new technologies. Building on this, programs ought to go beyond mere functional training by incorporating critical, reflective discussions about the ethical, professional, and sociotechnical aspects of AI. These conversations are likely more effective in addressing the ongoing anxieties highlighted in this study. Additionally, the research emphasizes the need for statistical literacy as a foundation for the responsible use of AI tools in research. Teacher education should therefore combine AI training with basic instruction in research methods and data analysis to prevent over-dependence on AI as an opaque “black box” and to enable future educators to use these tools thoughtfully and proficiently.

Future research should build on these insights by addressing the study’s limitations. Given that AI anxiety and dispositional traits, such as resistance to change, remained stable over the 14-week intervention, longitudinal studies are needed to determine whether prolonged exposure over multiple semesters can produce more significant changes. Future research should employ experimental or quasi-experimental designs with control groups to establish a more definitive causal link between AI-focused interventions and the observed outcomes. Comparing different instructional approaches or interventions across various teacher populations would also yield more generalizable insights and a deeper understanding of how multiple educators respond to AI interventions. Moreover, research across different subject areas could identify discipline-specific best practices for integrating AI. Additionally, future work could integrate rubric performance as an external validity check (e.g., correlating rubric totals with post-course acceptance), provided sample size permits.

## Limitations

This study employed a single-group pre-test and post-test methodological framework within a mandated, semester-long academic course. While this design was necessitated by institutional and ethical limitations that precluded withholding AI-supported instruction, the absence of a comparative group limits the capacity to ascertain causal relationships. Differences identified between pre-test and post-test might be connected to developmental changes, external circumstances (including the dynamic dialogue regarding AI), or the effects of repeated assessments. Given that the same instructor facilitated and assessed the course, instructor-related effects cannot be dismissed. Consequently, the findings should be interpreted as correlational rather than indicative of causation. In subsequent iterations of the course, pre-registration will be implemented, and whenever feasible, a control group or comparative cohort will be incorporated to enhance the robustness of the evaluation.

All outcomes were derived from self-reported Likert-type tools, which are at risk of social desirability bias, demand characteristics, and common-method bias. Although the internal consistency was deemed satisfactory, longitudinal measurement invariance was not assessed; therefore, some of the observed changes may represent shifts in response rather than genuine differences in scores. Future research should triangulate survey data with (a) behavioral telemetry of AI utilization (e.g., duration of engagement, prompt frequency, feature usage), (b) blinded rubric-based assessments of student submissions, (c) peer or instructor evaluations of observable conduct, and (d) qualitative interviews or focus groups to elucidate the underlying mechanisms.

The external validity of the findings is constrained. The study involved a specific group of pre-service teachers from a single institution enrolled in a 14-week course that featured AI tools within project-centered activities. Its generalizability to other contexts, disciplines, or research methodologies remains ambiguous. Future investigations should employ matched or staggered comparisons, analyze dose–response relationships utilizing verified usage logs, and extend follow-up durations to assess the sustainability of attitudinal and acceptance changes.

The course emphasized skill-based engagement rather than affective or ethical contemplation. The absence of components aimed at reducing anxiety, such as debates, reflective journaling, or values clarification, may elucidate the lack of impact on AI-related anxiety. While attitudes toward AI and AI acceptance were analyzed concurrently, discriminant validity (e.g., confirmatory factor analysis) was not evaluated. Future studies should incorporate these diagnostic assessments and behavioral indicators to mitigate common-method bias.

## Supplementary Information

Supplementary Material 1.

Supplementary Material 2.

Supplementary Material 3.

## Data Availability

The dataset underpinning this study is openly available in the Harvard Dataverse (Social Science Repository) at [10.7910/DVN/P7YOW3](10.7910/DVN/P7YOW3). All data are de-identified and include pre- and post-test scores and a codebook; no personally identifying information is included.
