# Supplementary material for: The impact of AI integration in project preparation in education course on pre-service teachers’ innovativeness, AI anxiety, attitudes, and acceptance
Source: BMC Psychol. 2025 Nov 24;13:1297. doi: 10.1186/s40359-025-03647-3 (PMC12642211; doi:10.1186/s40359-025-03647-3)
Supplement: Supplementary file 1 — Supplementary Material 1. [file 40359_2025_3647_MOESM1_ESM.docx]

**Adapted Rubric for Research Project Evaluation**

| **Section** | **Criterion** | **1 (Inadequate)** | **3 (Moderate)** | **5 (Excellent)** |
| --- | --- | --- | --- | --- |
| INTRODUCTION | Problem Definition & Significance | The problem is unclear; there is no clear statement about why the research is important. | The problem is partially defined, but its significance is only described in general terms. | The problem is clearly articulated, its scope is well-defined, and the importance and contribution of the research are strongly emphasized. |
|  | Research Purpose/Question (Hypothesis) | The purpose or research question is unclear or not mentioned at all. | The purpose/question is generally outlined, but the hypothesis and expected outcomes are not sufficiently clear. | The purpose/question is clearly stated, with explicit hypotheses or assumptions; the expected contribution and outcomes are understandable. |
| LITERATURE REVIEW | Validity & Variety of Sources | Very few or unreliable sources are used. | The sources are somewhat reliable, but there are gaps in terms of recency and variety. | The sources are up-to-date, diversified (books, articles, reports, etc.), and comprehensively include leading research in the field. |
|  | Critical Evaluation of the Literature | Citations and summaries are superficial, and previous studies are not adequately analyzed. | The sources are summarized, but there is only a partial or superficial critical perspective or comparison. | Similar and different studies in the literature are thoroughly compared, with critical commentary and research gaps clearly identified. |
| METHODOLOGY | Research Design & Rationale | It is unclear which method was used and why, with no explanation provided. | The method is mentioned, but there is insufficient detail about its rationale or alignment with the research design. | The research design (quantitative, qualitative, mixed, etc.) is clear, and the reasoning for selecting this method and how it aligns with the research objectives is convincingly explained. |
|  | Data Collection & Analysis Process | Data collection methods are vague or lack reliability, and the analysis process is not explained. | Data collection is outlined in general terms, but the analysis technique or statistical methods are only partially explained, lacking sufficient detail. | Data collection tools (surveys, interviews, observations, etc.) and analysis methods (statistical analysis, content analysis, etc.) are clearly and thoroughly described; measures to ensure validity are specified. |
| FINDINGS | Data Presentation | The findings are disorganized, hard to understand, or supported by inadequate tables/graphs. | The findings are somewhat understandable, but the visual materials (tables, graphs) are not sufficiently explained or are limited. | The findings are presented systematically, using appropriate tables and graphs; a structure is established for easy comprehension, and additional visuals are effectively used. |
|  | Clarity & Consistency of Findings | The findings do not adequately answer the research question; there is missing or weak data to support conclusions. | The findings partially align with the research question but lack depth or thoroughness. | The findings directly address the research question with clear and concrete data; the interpretation and analysis are consistent and understandable. |
| DISCUSSION | Interpretation of Findings | There is minimal or superficial commentary on the findings, with no link to the literature. | The findings are interpreted to some extent, but the connection to the literature is partial or superficial. | The findings are interpreted in depth and thoroughly compared to previous studies; similarities and differences are clearly highlighted. |
|  | Limitations & Recommendations for Future Research | These are not mentioned at all or are addressed only in very general terms. | Some limitations and possible future research directions are mentioned but only superficially. | The study’s limitations are clearly stated, with concrete and detailed suggestions for future research; alternative methods and research opportunities are extensively discussed. |
| CONCLUSION | Overall Summary & Emphasis of Conclusions | The study’s conclusions are not clearly stated; it is unclear what the main takeaway is. | Conclusions are mentioned, but it is not clear how they fulfill the main research goal or hypothesis. | Conclusions are directly tied to the research question and objectives, with the study’s main contributions and implications strongly emphasized. |
|  | Practical & Theoretical Contributions | Contributions are not discussed at all or remain very abstract. | Theoretical or practical contributions are partially stated but lack concrete examples or specifics. | The findings are explained both theoretically and practically, indicating how they can be applied, which fields they contribute to, and what new directions they might inspire, supplemented with clear examples. |
|  | Overall Writing Quality & Readiness for Publication | The text contains numerous writing errors; the academic style is weak, and there is a lack of coherence in the research. | The text is generally understandable, but there are deficiencies in writing style, citation format, or academic conventions. | The text is coherent, well-structured, and written in a suitable academic style; citations, references, and formatting rules are applied consistently, with proper editing and reviewing efforts demonstrated. |
