# Supplementary material for: The impact of AI integration in project preparation in education course on pre-service teachers’ innovativeness, AI anxiety, attitudes, and acceptance
Source: BMC Psychol. 2025 Nov 24;13:1297. doi: 10.1186/s40359-025-03647-3 (PMC12642211; doi:10.1186/s40359-025-03647-3)
Supplement: Supplementary file 2 — Supplementary Material 2. [file 40359_2025_3647_MOESM2_ESM.docx]

## Appendix

### Weekly AI-Integrated Learning Activities, Exemplar Prompts (English–Turkish), and Tools Used

| **Week** | **Main Focus** | **AI Tools Commonly Used** | **Example Prompts / Örnek Yönlendirmeler** |
| --- | --- | --- | --- |
| **3** | **Research Question Development**  (Araştırma Sorusu Geliştirme) | ChatGPT Plus, Gemini | **EN:** “Generate 5 research questions for a [qualitative or quantitative] study on *[your topic]* and present them in APA 7 format.”  **TR:** “*[Araştırma konun]* ile ilgili nitel veya nicel bir çalışma için 5 araştırma sorusu üret ve bunları APA 7 biçiminde sun.”  **EN:** “Generate 3–5 open-ended qualitative research questions on *[your topic]* in APA 7 style.”  **TR:** “*[Araştırma konun]* ile ilgili 3–5 açık uçlu nitel araştırma sorusu oluştur ve APA 7 biçiminde yaz.” |
| **4** | **Introduction Writing**  (Giriş Bölümü Yazımı) | ChatGPT Plus, Perplexity | **EN:** “Write a 150-word introduction explaining the importance of *[your topic]* in education, formatted according to APA 7 style.”  **TR:** “Eğitim alanında *[araştırma konun]* önemini açıklayan, APA 7 biçimine uygun 150 kelimelik bir giriş paragrafı yaz.” |
| **5** | **Literature Review**  (Alan Yazın Taraması) | Elicit, Consensus, NotebookLM | **EN:** “Summarize 5 recent (2020–2024) peer-reviewed studies on *[your construct]* and format citations in APA 7 style.”  **TR:** “2020–2024 arasında yayınlanan *[değişken]* konulu 5 hakemli çalışmayı özetle ve APA 7 biçiminde kaynaklandır.”  **EN:** “Write a synthesis paragraph linking *[construct A]* and *[construct B]* in APA 7 style.”  **TR:** “*[Değişken A]* ile *[Değişken B]* arasındaki ilişkiyi özetleyen bir sentez paragrafı oluştur (APA 7 biçiminde).” |
| **6** | **Research Methodology** (Araştırma Yöntemi) | ChatGPT Plus, Gemini | **EN:** “Write a Method section for a *[quantitative or qualitative]* study on *[your topic]* describing participants, tools, and procedure in APA 7 format.”  **TR:** “*[Araştırma konun]* için nitel veya nicel bir çalışmaya yönelik katılımcıları, araçları ve süreci açıklayan bir Yöntem bölümü yaz (APA 7 biçiminde).”  **EN:** “Write a qualitative method section including sampling strategy, data collection steps, and analysis plan in APA 7 style.”  **TR:** “Örnekleme stratejisi, veri toplama adımları ve analiz planını içeren bir nitel yöntem bölümü yaz (APA 7 biçiminde).” |
| **7** | **Data Collection**  (Veri Toplama) | ChatGPT Plus, Gemini, NotebookLM | **Quantitative Studies:**  **EN:** “Describe how *[the validated questionnaire]* was administered and summarize its subdimensions and scoring procedure in APA 7 academic style.”  **TR:** “*[Geçerliği sağlanmış ölçeğin]* nasıl uygulandığını açıkla ve alt boyutlarını ile puanlama sürecini APA 7 akademik biçiminde özetle.”  **Qualitative Studies:**  **EN:** “Create 8–10 semi-structured interview questions for *[your topic]* and write an APA 7-formatted interview protocol.”  **TR:** “*[Araştırma konun]* için 8–10 yarı yapılandırılmış görüşme sorusu oluştur ve APA 7 biçiminde bir görüşme yönergesi hazırla.”  **EN:** “Summarize ethical considerations and consent procedures for interviews in APA 7 style.”  **TR:** “Görüşmeler için etik hususları ve onam süreçlerini APA 7 biçiminde özetle.” |
| **8** | **Data Analysis**  (Veri Analizi) | ChatGPT Plus, Gemini | **Quantitative:**  **EN:** “Analyze my dataset exported from Excel. Compute descriptive statistics (mean, SD, correlations) for the variables below and interpret the results in APA 7 Results section format.”  **TR:** “Excel’den dışa aktardığım veri setini analiz et. Aşağıdaki değişkenler için betimsel istatistikleri (ortalama, SS, korelasyon) hesapla ve sonuçları APA 7 Bulgular bölümü biçiminde yorumla.”  **Qualitative:**  **EN:** “Analyze the interview data pasted below. Identify recurring codes and themes, then write the Results section in APA 7 narrative style with supporting quotes.”  **TR:** “Aşağıya yapıştırdığım görüşme verilerini analiz et. Tekrarlayan kod ve temaları belirle, sonra destekleyici alıntılarla birlikte APA 7 anlatım biçiminde Bulgular bölümünü yaz.”  **EN:** “Create a codebook table listing theme names, definitions, and sample excerpts in APA 7 table format.”  **TR:** “Tema adlarını, tanımlarını ve örnek alıntıları içeren bir kod tablosu oluştur (APA 7 tablo biçiminde).” |
| **9** | **Data Reporting** (Bulguların Raporlanması) | ChatGPT Plus | **EN:** “Write the Results section for *[your study]* using the data below. Present means, SDs, or themes in APA 7 format.”  **TR:** “*[Çalışman]* için aşağıdaki verileri kullanarak Bulgular bölümünü yaz. Ortalamaları, SS veya temaları APA 7 biçiminde sun.”  **EN:** “Integrate direct participant quotes from *[your interviews]* into an APA 7-formatted Results section.”  **TR:** “*[Görüşme verilerinden]* doğrudan katılımcı alıntılarını APA 7 biçiminde Bulgular bölümüne ekle.” |
| **10** | **Discussion**  (Tartışma Bölümü) | ChatGPT Plus, Gemini | **EN:** “Write a discussion paragraph interpreting *[your key finding]* in relation to previous studies (APA 7 style).”  **TR:** “*[Temel bulgun]*u önceki çalışmalarla ilişkilendirerek yorumlayan bir tartışma paragrafı yaz (APA 7 biçiminde).”  **EN:** “Write three practical implications for *[your field]* based on the results (APA 7 discussion style).”  **TR:** “Sonuçlara dayanarak *[alanın]* için üç uygulama çıkarımı yaz (APA 7 tartışma biçiminde).” |
| **11** | **Draft Revision** (Taslak Revizyonu) | ChatGPT Plus | **EN:** “Revise the paragraph below to improve clarity, cohesion, and APA 7 academic tone.”  **TR:** “Aşağıdaki paragrafı açıklık, bütünlük ve APA 7 akademik üslubu açısından geliştir.” |
| **12** | **Autonomous Editing**  (Bağımsız Düzenleme) | ChatGPT Plus, NotebookLM | **EN:** “Review my Discussion section for coherence, APA 7 compliance, and redundancy; suggest specific improvements.”  **TR:** “Tartışma bölümümü bütünlük, APA 7 uygunluğu ve tekrarlar açısından değerlendir; somut geliştirme önerileri sun.”  **EN:** “Edit my Conclusion section to align with APA 7 academic conventions.”  **TR:** “Sonuç bölümümü APA 7 akademik yazım kurallarına uygun hale getir.” |
| **13-14** | **Manuscript Finalization** (Makale Tamamlama) | ChatGPT Plus, Perplexity | **EN:** “Check the entire manuscript for APA 7 format consistency (headings, tables, citations, references).”  **TR:** “Makalenin tamamını (başlıklar, tablolar, atıflar, kaynakça) APA 7 biçim tutarlılığı açısından kontrol et.”  **EN:** “Write a 100-word APA 7 abstract summarizing *[your study]*.”  **TR:** “*[Çalışmanı]* özetleyen 100 kelimelik bir APA 7 öz yaz.” |
