# Supplementary material for: The impact of AI integration in project preparation in education course on pre-service teachers’ innovativeness, AI anxiety, attitudes, and acceptance
Source: BMC Psychol. 2025 Nov 24;13:1297. doi: 10.1186/s40359-025-03647-3 (PMC12642211; doi:10.1186/s40359-025-03647-3)
Supplement: Supplementary file 3 — Supplementary Material 3. [file 40359_2025_3647_MOESM3_ESM.docx]

## Appendix

### Pearson correlations among the four primary constructs and their subdimensions at pre-test and post-test

Supplement 1 shows Pearson correlations among the four primary constructs and their subdimensions at pre-test.

### Supplement 1:

|  | **Variable**  **(Pre-test)** | **1** | **2** | **3** | **4** | **5** | **6** | **7** | **8** | **9** | **10** | **11** | **12** | **13** | **14** | **15** | **16** | **17** | **18** |
| --- | --- | --- | --- | --- | --- | --- | --- | --- | --- | --- | --- | --- | --- | --- | --- | --- | --- | --- | --- |
| 1 | Acceptance (Total) | - |  |  |  |  |  |  |  |  |  |  |  |  |  |  |  |  |  |
| 2 | Performance Expectancy | 0.86*** | - |  |  |  |  |  |  |  |  |  |  |  |  |  |  |  |  |
| 3 | Effort Expectancy | 0.72*** | 0.56*** | - |  |  |  |  |  |  |  |  |  |  |  |  |  |  |  |
| 4 | Social Expectancy | 0.81*** | 0.53** | 0.32 | - |  |  |  |  |  |  |  |  |  |  |  |  |  |  |
| 5 | Facilitating Expectancy | 0.84*** | 0.69*** | 0.45** | 0.71*** | - |  |  |  |  |  |  |  |  |  |  |  |  |  |
| 6 | AI Anxiety (Total) | -0.47** | -0.44** | -0.43* | -0.25 | -0.41* | - |  |  |  |  |  |  |  |  |  |  |  |  |
| 7 | Job Replacement | -0.36* | -0.32 | -0.21 | -0.29 | -0.37* | 0.91*** | - |  |  |  |  |  |  |  |  |  |  |  |
| 8 | Sociotechnical Blindness | -0.44** | -0.40* | -0.36* | -0.28 | -0.44** | 0.89*** | 0.80*** | - |  |  |  |  |  |  |  |  |  |  |
| 9 | AI Configuration | -0.34 | -0.22 | -0.30 | -0.29 | -0.27 | 0.84*** | 0.86*** | 0.72*** | - |  |  |  |  |  |  |  |  |  |
| 10 | Learning | -0.43* | -0.40* | -0.30 | -0.27 | -0.46** | 0.93*** | 0.91*** | 0.81*** | 0.84*** | - |  |  |  |  |  |  |  |  |
| 11 | AI Attitudes (Total) | 0.84*** | 0.75*** | 0.50** | 0.72*** | 0.78*** | -0.55*** | -0.49** | -0.55*** | -0.35* | -0.51** | - |  |  |  |  |  |  |  |
| 12 | Cognitive | 0.79*** | 0.72*** | 0.39* | 0.71*** | 0.74*** | -0.41* | -0.39* | -0.41* | -0.28 | -0.37* | 0.89*** | - |  |  |  |  |  |  |
| 13 | Affective | 0.82*** | 0.74*** | 0.48** | 0.69*** | 0.75*** | -0.55*** | -0.50** | -0.57*** | -0.35* | -0.52** | 0.98*** | 0.84*** | - |  |  |  |  |  |
| 14 | Behavioral | 0.83*** | 0.72*** | 0.52** | 0.70*** | 0.76*** | -0.54*** | -0.47** | -0.54*** | -0.35* | -0.51** | 0.99*** | 0.84*** | 0.95*** | - |  |  |  |  |
| 15 | Individual Innovativeness (Total) | 0.26 | 0.14 | -0.01 | 0.46** | 0.20 | 0.30 | 0.17 | 0.28 | 0.12 | 0.14 | 0.32 | 0.30 | 0.28 | 0.33 | - |  |  |  |
| 16 | Resistance to Change | -0.10 | -0.17 | -0.23 | 0.15 | -0.12 | 0.21 | 0.05 | 0.11 | -0.07 | 0.16 | -0.17 | -0.08 | -0.16 | -0.20 | 0.34* | - |  |  |
| 17 | Opinion Leading | 0.22 | 0.14 | 0.17 | 0.20 | 0.25 | 0.14 | 0.17 | 0.19 | 0.14 | 0.02 | 0.31 | 0.20 | 0.26 | 0.36* | 0.55*** | -0.48** | - |  |
| 18 | Openness to Experience | 0.20 | 0.22 | 0.12 | 0.16 | 0.12 | -0.02 | -0.04 | 0.05 | 0.06 | -0.09 | 0.31 | 0.21 | 0.29 | 0.34* | 0.50** | -0.43* | 0.59*** | - |
| 19 | Risk Taking | 0.41* | 0.39* | 0.14 | 0.42* | 0.34* | 0.10 | 0.12 | 0.14 | 0.21 | 0.06 | 0.50** | 0.51** | 0.47** | 0.48** | 0.48** | -0.29 | 0.43* | 0.40* |

Note. N *=* 34. Values are Pearson’s r, two-tailed. *p <0.05, ** p <0.01***, ******p **<0.001.**

### Supplement 2:

Supplement 2 shows Pearson correlations among the four primary constructs and their subdimensions at post-test

|  | **Variable**  **(Post-test)** | **1** | **2** | **3** | **4** | **5** | **6** | **7** | **8** | **9** | **10** | **11** | **12** | **13** | **14** | **15** | **16** | **17** | **18** |
| --- | --- | --- | --- | --- | --- | --- | --- | --- | --- | --- | --- | --- | --- | --- | --- | --- | --- | --- | --- |
| 1 | Acceptance (Total) | - |  |  |  |  |  |  |  |  |  |  |  |  |  |  |  |  |  |
| 2 | Performance Expectancy | 0.93*** | - |  |  |  |  |  |  |  |  |  |  |  |  |  |  |  |  |
| 3 | Effort Expectancy | 0.85*** | 0.78*** | - |  |  |  |  |  |  |  |  |  |  |  |  |  |  |  |
| 4 | Social Expectancy | 0.85*** | 0.68*** | 0.52** | - |  |  |  |  |  |  |  |  |  |  |  |  |  |  |
| 5 | Facilitating Expectancy | 0.87*** | 0.75*** | 0.61*** | 0.83*** | - |  |  |  |  |  |  |  |  |  |  |  |  |  |
| 6 | AI Anxiety (Total) | -0.54*** | -0.57*** | -0.58*** | -0.32 | -0.38* | - |  |  |  |  |  |  |  |  |  |  |  |  |
| 7 | Job Replacement | -0.50** | -0.47** | -0.50** | -0.36* | -0.42* | 0.89*** | - |  |  |  |  |  |  |  |  |  |  |  |
| 8 | Sociotechnical Blindness | -0.42* | -0.48** | -0.43* | -0.21 | -0.31 | 0.93*** | 0.80*** | - |  |  |  |  |  |  |  |  |  |  |
| 9 | AI Configuration | -0.53** | -0.49** | -0.52** | -0.43* | -0.38* | 0.85*** | 0.80*** | 0.76*** | - |  |  |  |  |  |  |  |  |  |
| 10 | Learning | -0.58*** | -0.58*** | -0.57*** | -0.39* | -0.47** | 0.92*** | 0.87*** | 0.84*** | 0.76*** | - |  |  |  |  |  |  |  |  |
| 11 | AI Attitudes (Total) | 0.78*** | 0.83*** | 0.63*** | 0.58*** | 0.66*** | -0.66*** | -0.65*** | -0.55*** | -0.54** | -0.69*** | - |  |  |  |  |  |  |  |
| 12 | Cognitive | 0.67*** | 0.74*** | 0.55*** | 0.49** | 0.47** | -0.59*** | -0.55*** | -0.49** | -0.48** | -0.56*** | 0.87*** | - |  |  |  |  |  |  |
| 13 | Affective | 0.78*** | 0.84*** | 0.65*** | 0.57*** | 0.63*** | -0.62*** | -0.61*** | -0.49** | -0.51** | -0.66*** | 0.98*** | 0.84*** | - |  |  |  |  |  |
| 14 | Behavioral | 0.74*** | 0.78*** | 0.58*** | 0.55*** | 0.69*** | -0.66*** | -0.67*** | -0.57*** | -0.53** | -0.70*** | 0.97*** | 0.78*** | 0.91*** | - |  |  |  |  |
| 15 | Individual Innovativeness (Total) | 0.11 | 0.00 | -0.05 | 0.28 | 0.23 | 0.19 | 0.06 | 0.21 | -0.07 | 0.19 | -0.07 | -0.01 | -0.10 | -0.05 | - |  |  |  |
| 16 | Resistance to Change | -0.22 | -0.33 | -0.31 | 0.04 | -0.10 | 0.47** | 0.35* | 0.40* | 0.17 | 0.42* | -0.46** | -0.42* | -0.46** | -0.43* | 0.51** | - |  |  |
| 17 | Opinion Leading | 0.28 | 0.25 | 0.24 | 0.21 | 0.34* | -0.17 | -0.25 | -0.05 | -0.16 | -0.17 | 0.37* | 0.29 | 0.38* | 0.36* | 0.38* | -0.43* | - |  |
| 18 | Openness to Experience | 0.27 | 0.28 | 0.23 | 0.23 | 0.17 | -0.30 | -0.29 | -0.17 | -0.27 | -0.22 | 0.31 | 0.43* | 0.24 | 0.30 | 0.32 | -0.48** | 0.44** | - |
| 19 | Risk Taking | 0.17 | 0.25 | 0.12 | 0.05 | 0.21 | -0.19 | -0.12 | -0.33 | -0.13 | -0.17 | 0.19 | 0.21 | 0.15 | 0.20 | 0.37* | -0.22 | 0.21 | 0.34* |

Note. N *=* 34. Values are Pearson’s r, two-tailed. *p <0.05, ** p <0.01***,* *****p **<0.001.**
